# Bilateral Repetitive Transcranial Magnetic Stimulation With the H-Coil in Parkinson's Disease: A Randomized, Sham-Controlled Study

**DOI:** 10.3389/fneur.2020.584713

**Published:** 2021-02-18

**Authors:** Francesca Spagnolo, Mario Fichera, Raffaella Chieffo, Gloria Dalla Costa, Marco Pisa, Maria Antonietta Volonté, Monica Falautano, Abraham Zangen, Giancarlo Comi, Letizia Leocani

**Affiliations:** ^1^Experimental Neurophysiology Unit, Institute of Experimental Neurology - INSPE, Scientific Institute Hospital San Raffaele, Milan, Italy; ^2^San Raffaele Vita-Salute University, Milan, Italy; ^3^Neurological Department, Hospital San Raffaele, Milan, Italy; ^4^Neuropsychology and Clinical Psychology Service, Hospital San Raffaele, Milan, Italy; ^5^Neuroscience Laboratory, Ben-Gurion University of the Negev, Beer Sheva, Israel

**Keywords:** rTMS (repetitive transcranial magnetic stimulation), H-coil = hesed coil, motor cortex, movement disorder, non-invasive brain stimulation (NIBS)

## Abstract

**Background:** Pilot open-label application of high-frequency repetitive transcranial magnetic stimulation (rTMS) with H-coil in Parkinson's Disease (PD) have shown promising results.

**Objective:** To evaluate safety and efficacy of high-frequency rTMS with H-coil in PD in a double-blind, placebo-controlled, randomized study.

**Methods:** Sixty patients with PD were randomized into 3 groups: M1-PFC (real stimulation on primary motor-M1 and pre-frontal cortices-PFC), M1 (real rTMS on M1, sham on PFC), Sham (apparent stimulation). Primary outcome was baseline-normalized percent improvement in UPDRS part III OFF-therapy at the end of treatment (12 rTMS sessions, 4 weeks). Secondary outcomes were improvement in UPDRS part III sub-scores, timed tests, and neuropsychological tests. Statistical analysis compared improvement following real and sham stimulation at the end of the protocol using either a *t*-test or a Mann-Whitney test.

**Results:** All patients tolerated the treatment and concluded the study. One patient from M1-PFC group was excluded from the analysis due to newly discovered uncontrolled diabetes mellitus. No serious adverse effect was recorded. At the end of treatment, patients receiving real rTMS (M1-PFC and M1 combined) showed significantly greater improvement compared to sham in UPDRS part III total score (*p* = 0.007), tremor subscore (*p* = 0.011), and lateralized sub-scores (*p* = 0.042 for the more affected side; *p* = 0.012 for the less affected side). No significant differences have been oserved in safety and efficacy outcomes between the two real rTMS groups. Notably, mild, not-distressing and transient dyskinesias occurred in 3 patients after real rTMS in OFF state.

**Conclusions:** The present findings suggest that high-frequency rTMS with H-coil is a safe and potentially effective procedure and prompt larger studies for validation as add-on treatment in PD.

## Introduction

The development of invasive therapeutic options such as deep brain stimulation has opened new perspectives but remains a niche procedure (1). In that regard, non-invasive neuromodulation techniques, such as repetitive transcranial magnetic stimulation (rTMS), have the potential to play a role in Parkinson's disease (PD) therapy (2). Contrasting results have been reported after rTMS over primary (M1) and non-primary motor cortex (i.e., supplementary motor area-SMA and pre-frontal cortex-PFC), ranging from improvement, to worsening (3, 4) of motor symptoms. The discrepancy among studies can be partially related to differences in stimulation parameters and targets (2, 5). However, a 2018 meta-analysis of placebo-controlled trials found a significant beneficial effect on motor symptoms of high-, but not low-frequency rTMS (5, 6). More recently, combined approach with low-frequency M1 stimulation and high-frequency PFC stimulation with H-coil did not find an advantage for real treatment over sham (7). rTMS has been mostly applied using the standard circular or figure-of-8 coils, which act on relatively narrow cortical regions. The H-coil has been designed to stimulate a wider area of effective cortical stimulation compared with the standard coils (8); considering the widespread cortical dysfunction in PD (9), this could be seen as a possible advantage and not as a limitation. In fact, positive results have been achieved in PD using the focal coil with sequential bilateral stimulation of M1, and possibly associating M1 and DLPFCstimulation (2, 5, 10–12). Moreover, an open-label pilot study performed by our group showed that high-frequency rTMS stimulation of bilateral M1 and PFC with H-coil might serve as a safe and effective treatment for PD (13). In the current study we aimed at further exploring the therapeutic effects and safety profile of rTMS with H coil on PD motor symptoms in a double-blind, placebo-controlled trial. Moreover, we tested the effects the effects of M1 stimulation alone.

## Patients and Methods

### Patients

Sixty patients suffering from idiopathic PD according to United Kingdom PD Brain Bank criteria were recruited in our center. The protocol was approved by the local ethics committee and all patients gave their written consent prior to enrolment; an institutional study monitoring was provided as well. Inclusion criteria were: age <80 years, Hoehn and Yahr (HY) scale II-IV, stable anti-depressive, and anti-parkinsonian therapy for at least 2 months prior to enrollment, and ability to provide oral and written informed consent. Patients were excluded if they had other medical, psychiatric or neurological disorders or any contraindication to TMS (uncontrolled hypertension, history of seizures, recent head trauma, presence of metal implants, etc.). Medications were kept constant throughout the trial, and interventions and evaluations were performed at the same time of the day for each patient. For analysis, dosages of antiparkinsonian medications were expressed as the levodopa equivalent daily dose (LEDD) (14). Clinical-demographical data are shown in Table 1.

**Table 1:** Baseline characteristics for patients enrolled.

**Group**	**Age**	**Sex**	**Disease duration**	**LEDD**	**MDS-UPDRS III**	**H&Y stage**	**WS**	**BS**	**Axial**	**Tremor**	**Rigidity**
**Levels**	**Mean years (SD)**	**M/F**	**Mean years (SD)**	**Mean mg (SD)**	**Mean score (SD)**	**Median score (IQR)**	**Mean score (SD)**	**Mean score (SD)**	**Median score (IQR)**	**Mean score (SD)**	**Mean score (SD)**
Real (*n* = 39)	62 (9)	27/12	6.7 (3.8)	627 (317)	40.7 (10.6)	2.0 (2.0–2.0)	17.0 (4)	11.2 (4.3)	8.0 (6.0–10.0)	8.5 (5.1)	7.7 (3.5)
M1-PFC (*n* = 19)	63.9 (10)	12/7	7.6 (4.9)	585.1 (304)	42.4 (11.2)	2.0 (2.0–2.5)	17.5 (3.3)	11.8 (4.3)	8.0 (6.5–11.0)	7.3 (3.9)	8.5 (2.9)
M1 (*n* = 20)	60.4 (8.1)	15/5	5.8 (2.1)	666.9 (332)	39.1 (10)	2.0 (2.0–2.0)	16.7 (4.7)	10.7 (4.4)	8.0 (6.0–10.0)	9.7 (5.9)	7.1 (4)
Sham (*n* = 20)	64.2 (5.5)	14/6	7.2 (3)	629.2 (315)	43.3 (9)	2.0 (2.0–2.0)	17.1 (4)	12.2 (3.1)	8.0 (4.0–10.5)	6.6 (3.2)	8.5 (3.7)
*P*	0.36		0.65	0.98	0.34	0.14	0.94	0.38	0.04	0.14	0.41

*LEDD, Levodopa Equivalent Daily Dose; UPDRSIII, Unified Parkinson's Disease Rating Scale part III; H&Y, Hoen and Yahr stage; WS, worse side subscore; BS, better side subscore; SD, standard deviation; IQR, interquartile range*.

### Study Design

**Figure 1:**
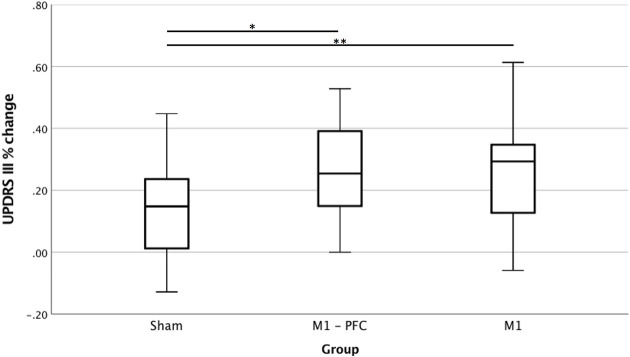
Percent variation of Unified Parkinson's Disease rating scale (UPDRS) part III at T2 compared to basal. Real group is obtained by merging M1-PFC and M1 together. *T*-test, ^*^p < 0.05; ^**^p < 0.01. Positive values indicate improvement.

This study was a phase II randomized, double-blind trial with a parallel 3 arms design (clinicaltrials.gov:NCT04638777). Two cortical areas, the primary motor cortex and the PFC, were chosen as targets based on a literature review and our previous experience (13). Thus, following inclusion and initial evaluation, participants were assigned randomly to the 3 groups with a 1:1:1 allocation (Figure 1): Group 1: real rTMS over both targets (M1-PFC); Group 2: Real rTMS over M1 + sham rTMS over the PFC (M1); Group 3: sham stimulation over both targets (Sham). The total duration of the protocol was 8 weeks, a 4-week active phase where patients underwent 3 sessions of rTMS each week (12 sessions in total), and a follow-up 4 weeks after the last evaluation. In this occasion, patients were asked to come to the center under their usual pharmacological therapy (ON-drug) to limit their discomfort, as participants were all outpatients. The main outcome was improvement of the MDS-UPDRS (Unified Parkinson's Disease Rating Scale) part III (15), evaluated as percent variation at the end of the active phase compared to baseline. Motor evaluation was obtained OFF drug at baseline (T0), and after the last rTMS session (T2). An evaluation in ON condition, under usual dopaminergic treatment, was carried on after the 11th session (T1) and, as said, a follow-up visit was performed 4 weeks after the end of treatment in ON drug condition (T3). MDS-UPDRS motor score was further analyzed according to Parkinson's disease lateralization in worse side (WS) and better side (BS), through the sum for each hemi-body of items 3.3–3.8 and 3.15–3.17 (range 0–36). Axial involvement was independently considered as the sum of items 3.1–3.3a, 3.9–3.13, 3.17e (range 0–36). Rigidity (items 3.3a–e, range 0–20) and Tremor (items 3.15–3.18, range 0–40) scores were calculated as well. Additional tests (see also Supplementary Data) included: Timed tests included Hand Tapping (HT), Foot Tapping (FT), Walking Time (WT), Nine-hole Peg Test (NHPT). HT was assessed using a two-buttons keyboard, asking the patient to press the two buttons alternatively, using one hand at a time, as quickly as possible. Total number of taps achieved in 20″ was considered as the score. Similarly, FT required patients to rapidly move up and down each leg at a time, during a 20″ interval; only movements trespassing determinate amplitude (>15 cm) were considered as valid. WT was assessed asking the patients to walk in a corridor of 10 meters length for four times. In addition, a neuropsychological evaluation in ON state was performed at the screening visit and before the 11th session of rTMS: MMSE; Digit-span task; Verbal Fluencies Test; Frontal Assessment Battery (FAB), and Beck Depression Inventory (BDI-II).

### rTMS

Treatment with high-frequency rTMS was administered using the bilateral H5-coil (16). M1 was stimulated first, after M1 stimulation the coil was moved 6 cm forward and placed symmetrically over the scalp for the PFC treatment. For the M1 target, rTMS intensity was set at 90% of the RMT, while for the PFC stimulation stimulation intensity was raised to 100% RMT, 840 stimuli at 10 Hz were delivered for each target. Resting motor threshold (RMT) (17) was determined before each session using a double criterion to increase safety, e.g., by surface electromyographic recording on the abductor pollicis brevis (APB) and by visual inspection of the whole limb(s) after each stimulation. Although the H5-coil is designed to stimulate both M1 simultaneously (18), for RMT determination and for M1 stimulation it was slightly tilted until reaching the optimal position for stimulating the more affected M1. In this configuration, despite more affected M1 received the strongest stimulation, less affected M1 was stimulated as well. PFC stimulation, on the other side, provided bilateral stimulation of medial and lateral pre-frontal cortices, with no specific target area. With the exception of the first and last sessions, rTMS was performed ON drug considering the reported increased effect of rTMS in this condition (11). The first and last sessions were performed after overnight withdrawal of dopaminergic drugs in order to allow clinical evaluation in OFF state. After each session, participants were interviewed for rTMS-related adverse events (19). In order to obtain blindness, a magnetic card was used to activate the coil with real or sham settings (20). Sham stimulation was achieved by activating a different circuit with current flowing in opposite directions in each of the double wires of the H-coil, in order to obtain auditory and cutaneous sensations similar to that of real rTMS. In all other accounts, the stimulation paradigm followed that of the real group (including RMT measurements). To minimize any risk of unblinding affecting the clinical assessments, all study variables were measured by physicians other than the personnel dedicated to performing rTMS and recording side effects after each session.

### Statistical Analysis

In our prior open label study (13), a standardized difference of 1.6 was observed after treatment with rTMS at UPDRS. Therefore, 18 patients per group will allow to demonstrate a therapeutic effect of each treatment group compared with sham stimulation with 90% power using and a level of significance of 0.05 according to the Altman nomograms.

SPSS v. 17.0 software was used for statistical analyses. All data in the text are presented as mean ± standard deviation (SD) or median and interquartile range (IQR) according to the normality of the distribution; effects were considered statistically significant if a *p*-value <0.05 was found. Improvement in both the primary and the secondary motor variables was evaluated considering both absolute and percent variation between baseline (T0) and the last rTMS session (T2), as already used by other authors (21); depending on the normal distribution of the variables considered, either a *t*-test or a Mann-Whitney test was used. For the analyses, data from M1-PFC and M1 groups were joined to obtain a merged (Real) group, then the effects of real rTMS vs. placebo were tested in a hierarchical order. When a significant difference was found between Real and Sham groups, subsequent analyses were carried out in the following order, comparing first M1-PFC vs. Sham, then M1 vs. Sham, and finally M1-PFC vs. M1. Given that we run only pre-determined and hierarchical analyses, no multiple comparison adjustment was required (22).

## Results

There were no significant difference in baseline features between groups, except for axial score which was significantly higher in Sham compared to Merged group (10.8 ± 4.0 vs. 9.7 ± 4.6, *p* = 0.044) and was not further analyzed. All enrolled patients completed the study. One patient in M1-PFC was excluded from analysis due to newly discovered uncontrolled type II diabetes mellitus at the end of the T2.

### Safety

No serious adverse events occurred. A total of seven patients in Real group reported mild, self-limiting side effects at the end of rTMS, including face twitches, judged as due to ipsilateral peripheral facial nerve activation by the physician attending the stimulation (2 subjects), headache (1 subject), and dizziness (1 subject). In three patients (two patients in M1-PFC and one patient in M1), clinical examination performed OFF-drug at T2, showed the presence of involuntary movements, lasting about 15 min after the end of the stimulation, similar to those experienced by the patients when in the ON state. These dyskinetic movements were not distressing and patients were only partly aware of them.

No adverse events were recorded during the final safety evaluation, performed 4 weeks after the last rTMS stimulation.

### Efficacy

#### UPDRS III

Real group showed a significantly higher degree of improvement than Sham group at T2 (27 ± 16 vs. 15 ± 17, *t* 2.88; *p* = 0.007) which signify a large effect size (Cohen's D 0.73) (Figure 1 and Supplementary Table 1). In absolute terms, mean variation was 10.6 points in the Real and 6.50 points in Sham group. Following the hierarchical analysis, we found a significant difference in percent variation between M1-PFC and Sham groups (*t* 2.70; *p* = 0.009) and between M1 vs. Sham group (*t* 2.07; p = 0.045) in both cases with a large effect size (Cohen's D of 0.88 and 0.67, respectively). No significant differences were detected between M1-PFC and M1.

#### Lateralized Scores

A significant absolute and percent improvement was observed in Real vs. Sham group for both WS and BS, with similar percent variation between the two sides. Real group improved of 5.0 ± 2.9 points (29 ± 16%) on WS (Table 2), compared to 3.3 ± 3.4 points (19 ± 21%) of Sham group (*t* 2.06; *p* = 0.048 and *t* 2.61; *p* = 0.012 for absolute and percent variations, respectively). Similar considerations are valid for BS as well (*Z* −2.14; *p* = 0.039 and *Z* −2.50; *p* = 0.012 for absolute and percent variations, respectively).

**Table 2:** Lateralized UPDSR scores. Data are presented as mean (SD).

**UPRDS III-WS subscore**	**T0**	**T2**	**T0–T2**	**T0–T2%**
Real	17.1 (4)	12.1 (3.9)	5 (2.9)	29 (16)
M1-PFC	17.5 (3.3)	12.1 (3.5)	5.4 (3)	30 (17)
M1	16.7 (4.7)	12.2 (4.3)	4.6 (2.8)	28 (15)
Sham	17.1 (4)	13.8 (4.4)	3.3 (3.4)	19 (21)
*P*	-	-	0.04	0.04
**UPDRS III-BS SUBSCORE**				
Real	11.2 (4.3)	7.9 (4.1)	3.3 (2.7)	29 (35)
M1-PFC	11.8 (4.3)	7.8 (3.8)	3.9 (2.2)	34 (23)
M1	10.7 (4.4)	7.9 (4.4)	2.8 (3)	25 (43)
Sham	12.2 (3.1)	10.2 (3.4)	2 (2.3)	16 (17)
*P*			0.03	0.01

*UPDRS III lateralized score to the worse (WS) and better side (BS) at baseline and at T2 and absolute (T0–T2) and percentage (T0–T2%) change. Data are presented as mean (SD) unless specified. P-value refers to real vs. sham groups*.

Following the hierarchical order, statistically significant differences were observed for WS when testing both absolute and percent variations (*t* 2.10; *p* = 0.050 and *t* 2.36; *p* = 0.030) in M1-PFC vs. Sham. When examining BS we found substantial differences both in absolute (*Z* −2.42; *p* = 0.018) and in percent variation (*Z* −2.60; *p* = 0.009) between these two groups. Subsequent analyses of intergroup differences between M1 and Sham group and between M1-PFC and M1 groups showed no significant effects.

#### Tremor

**Figure 2:**
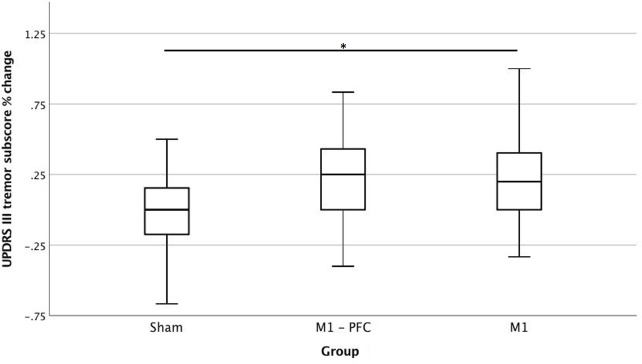
Percent variation of Unified Parkinson's Disease rating scale (UPDRS) part III tremor subscore at T2 compared to basal. Real group is obtained by merging M1-PFC and M1 together. *T*-test, ^*^p < 0.05. Positive values indicate improvement.

Data referring to Tremor subscores are shown in Figure 2 and Supplementary Table 2. In Sham group no significant variation occurred. Mean score for Real group improved from 8.5 at T0 to 6.3 at T2 (23%). A significant difference between Real and Sham group (*Z* −2.29; *p* = 0.022) variation emerged at T2. Significant differences were detected considering absolute variation, both for M1-PFC and M1 vs. sham, (*Z* −2.15; *p* = 0.019 and *Z* −2.15; *p* = 0.012, respectively). No significant difference between M1-PFC and M1 was found.

#### Rigidity

No significant difference was found at the end of treatment between Real and sham groups either for absolute or percent changes.

#### Timed Tests

Although rTMS stimulation targeted directly hand representation on M1, we also found a measurable improvement in FT scores of both sides. Mean number of taps for the WS increased from 27.8 to 35.8 in Real group reaching a statistically significant difference vs. Sham both in percent (*t* 2.27; *p* = 0.024) and absolute (*Z* −2.13; *p* = 0.031) variation of scores. For BS the difference in number of taps was not statistically significant when compared to sham. Regarding NHPT, at T2 mean time needed to complete the task for the WS was 4.0 s lower than basal in Real group (11% improvement), while placebo group improved only by 0.5 s (3% amelioration). A statistically significant difference between Real and sham groups was detected, both for absolute (*t* 2.10; *p* = 0.041) and percent (*t* 3.43; *p* = 0.001) variation at T2 compared to T0. Considering separately the two real groups, a substantial difference for percent variation at T2 emerged (*Z* −2.49; *p* = 0.009 and Z −2.54; *p* = 0.010 for M1-PFC and M1 vs. Sham, respectively). No significant differences were found between the two real groups (M1-PFC vs. M1). No other significant differences were found (See Also Supplementary Table 3).

#### Neuropsychological Testing

No significant group differences were detected for the neuropsychological variables (Supplementary Table 4).

#### Follow-Up

No significant difference between Real and Sham groups was identified when comparing variation between T1 and T3 (ON conditions) for both UPDRS III and timed tests. However, it has to be considered that the two groups were already different at T1 for the UPDRS III–ON (26,0 ± 9,0 for Real group vs. 33,5 ± 10,0 for Sham, *p* = 0.05), while there was no difference in raw UPDRS III scores at follow-up (27,0 ± 10,0 vs. 31,5 ± 8,3).

#### Correlations

No significant correlations were found between absolute and percent amelioration and clinical-demographic (age, basal UPDRS III, LEDD) characteristics in both real and sham groups.

## Discussion

This is the first placebo-controlled trial examining the effect of multiple treatment sessions with high-frequency rTMS with H-coil in Parkinson's disease. It extends our earlier open label, pilot research showing the safety and tolerability of 10 Hz rTMS with H-coil in Parkinson's disease (13). The present results suggest the efficacy of rTMS applied to motor and pre-frontal cortices in the treatment of motor symptoms in PD, with improvement in UPDRS part III (motor score) of 27% after active stimulation and a mean improvement of 15% after sham. The effects observed after active rTMS were not only statistically significant, but also clinically relevant: a total of 33 patients in Real group and 10 in Sham group met the criteria for a minimally clinically important improvement (23).

The present data are consistent with previous reports of positive effects of high-frequency rTMS applied over M1 alone (24) or in combination with pre-frontal stimulation (11) on motor symptoms in patients with Parkinson's disease; although the different coil used in our study and different parameter adopted may affect a direct comparison of the effects.

Compared to prior studies, the effect we found on motor symptoms, as evaluated through UPDRS III, reached higher values, similarly also placebo effect appears to be more pronounced (2, 9). The findings of this study differ from the work of Cohen and colleagues, where real treatment with combined low-frequency M1 stimulation and high-frequency PFC stimulation was not superior to sham stimulation (7), though a significant effect appeared in a preliminary open-label study of the same group (16). Despite recent meta-analysis favors low-frequency over high-frequency stimulation for motor symptoms control in PD (25), the effect could be different, even opposite, when considering the stimulation of single compared to multiple targets in the same session. The stimulation protocol we used for M1 stimulation is in agreement with prior literature suggesting that best results on motor symptoms are obtained with using high (vs low) frequency stimulation of bilater (vs unilateral) M1, and supporting the use of protocols with at least 18′000 pulses (5).

However, one of the major limits of this study is the absence of a direcly comparable follow-up to evaluate the long term effect of these stimulation protocol.

Although controversial, the pathophysiology of Parkinson's disease motor symptoms surely involve maladaptive, dysfunctional cortical and subcortical changes (26), as also demonstrated by neurophysiological studies (27). This hypothesis is strongly supported by studies in subthalamic nucleus deep brain stimulation (STN-DBS) implanted patients, showing how the clinical benefit in Parkinson's disease after DBS parallels its modification of pathological oscillatory pattern of cortico-basal ganglia loops (28). Such activity could be shared by rTMS, as suggested by studies in MPTP-treated monkeys (29) as well as in Parkinson's disease (30). With this respect, the simultaneous stimulation of several neural networks bilaterally could represent an advantage, although the present study was not designed to compare the impact of a simultaneous vs. sequential stimulation approach. Consistently, significant effects on motor scores have been reported after bilateral high-frequency M1 stimulation compared with sham, with no significant effect of left PFC stimulation alone or in addition to bilateral M1 (12).

Indeed, in the present study a significant improvement was found also in the less affected body side. Besides direct effects on M1, the present results could also depend on activation of non-primary motor areas (SMA and PMC) as well as pre-frontal areas, the second target of our stimulation.

We did not find significant differences between real M1 plus real PFC stimulation compared to real M1 plus sham PFC stimulation. However, the present study was not powered to compare the two active groups. Moreover, a direct comparison between M1 and PFC stimulation alone was not performed. It is also possible that the trend toward a higher treatment effect in the M1 plus PFC was rather an expression of a dose effect of the double stimulation rather than a site specific effect. Future studies should assess this issue.

However, the improvement observed in the BS, although present in both M1-PFC and M1 groups, was statistically significant only when comparing M1-PFC group vs. Sham, suggest that pre-frontal bilateral stimulation could have determined motor improvement to a greater extent in the BS than contralateral M1 stimulation alone, possibily due to the stimulation of non-primary motor areas on the corresponding hemispehere. Accordingly, the effect size of M1-PFC stimulation on motor symptoms tended to be slightly higher compared with M1 stimulation alone, despite not significantly so.

We did not find a significant advantage of rTMS compared with sham on walking time, which could be at least partly influenced by group dishomogeneity in axial scores already at baseline. The placebo effect found in the present study on several motor measures is consistent with previous reports (31). In PD, the expectation of a reward triggers a dopamine release not only in the nucleus accumbens but also in the nigrostriatal pathway (32), which may be associated with clinical improvement. Particular features such as the size and complexity of the machinery, the noise produced by the cooling system and the helmet-shaped H-coil may partly explain the the magnitude of the placebo effect found in our study (33).

In this study rTMS was delivered to patients while they were on medication, except for the first and last session. This allowed us to discover, in some patients undergoing real stimulation, slight, not-distressing, dyskinesias. A similar event was identified in our previous work (13) and, to our knowledge, has not been reported in other trials. This effect could be mediated by striatal medium spiny neurons through NMDA-mediated modulation of glutamatergic cortico-striatal fibers, as demonstrated in experimental models (34) or it could be secondary to dopamine release in the striatum. As of today, the exact mechanism behind dyskinesias is not fully understood and more information is needed to clarify this issue.

Examination of UPDRS part III tremor sub-score revealed a mean decrease of about 2.30 points in the real groups, while approximately no effect (mean decrease 0.20 points) was detected in the sham group at the end of treatment. Few other studies using rTMS have reported efficacy on the total amount of tremor. Siebner and colleagues (35) found a slight decrease (0.7 points) in tremor sub-score of UPDRS part III, mainly in the more affected hemibody contralateral to stimulation, while other authors did not find any important change in tremor sub-score (36). Tremor in PD has been linked with changes in the oscillatory pattern in the basal ganglia pathway, as some authors have found a distinctive neuronal oscillations pattern, linked to tremor, in the subthalamic nuclei in patients with PD (37). rTMS could have then helped to modulate abnormal oscillatory pattern by acting indirectly through motor cortex excitability.

No sure data exist so far about the duration of rTMS effects in neurologic patients. Few studies have demonstrated a long lasting effect of high frequency rTMS (7, 9, 38). The main limitation of our study is the lack of a proper follow-up. As patients had to come back to our Institute for the follow-up examination we limited the latter to safety data collection ON drugs, in order to minimize patient's discomfort and data loss, thus precluding the possibility to draw a conclusion about the duration of clinical efficacy on PD motor signs.

In conclusion, the possibility to offer a non-invasive neuromodulation treatment for Parkinson's disease treatment is a strong argument to further explore the effects of rTMS in this condition. The present results support and extend our understanding of the safety and efficacy profile of high-frequency bilateral rTMS with H-coil in the treatment of PD motor signs. Phase III studies with larger sample size are needed to further expand our knowledge on this approach, including medium-long term effects and to identify predictors markers of future individual response.

## Data Availability Statement

The raw data supporting the conclusions of this article will be made available by the authors, without undue reservation.

## Ethics Statement

The studies involving human participants were reviewed and approved by IRB Hospital San Raffaele. The patients/participants provided their written informed consent to participate in this study.

## Author Contributions

FS: study design, data collection, data analysis, and manuscript writing and revision. MFi and RC: data analysis and manuscript writing and revision. GDC and MP: data analysis and manuscript revision. MV: study design, clinical selection of patients, data interpretation, and manuscript revision for intellectual content. AZ: study design, data interpretation, and manuscript revision for intellectual content. GC: study design, supervision of data collection, study supervision, data interpretation, and manuscript revision for intellectual content. LL: study conception and design, supervision of data collection, and manuscript writing and revision. All authors contributed to the article and approved the submitted version.

## Conflict of Interest

AZ was a key inventor of the H-coil and acts as a consultant for Brainsway LTD. The remaining authors declare that the research was conducted in the absence of any commercial or financial relationships that could be construed as a potential conflict of interest.
