# Supplementary material for: Bilateral Repetitive Transcranial Magnetic Stimulation With the H-Coil in Parkinson's Disease: A Randomized, Sham-Controlled Study
Source: Front Neurol. 2021 Feb 18;11:584713. doi: 10.3389/fneur.2020.584713 (PMC7930321; doi:10.3389/fneur.2020.584713)
Supplement: Supplementary file 1 [file Data_Sheet_1.PDF]

Supplementary Data - Appendix

Timed Tests.

HT [1] was assessed using a two-buttons keyboard, asking the patient to press the two buttons alternatively, using one hand at a time, as quickly as possible. Total number of taps achieved in 20” was considered as the score. Similarly, FT required patients to rapidly move up and down each leg at a time, during a 20” interval; only movements trespassing determinate amplitude (> 15 cm) were considered as valid. TW was assessed asking the patients to walk in a corridor of 10 meters length for four times; travel time in seconds was used as score for this test. The NHPT consists in moving nine pegs within nine-holes located on a perforated board and then remove them [2]; the task was repeated two times and only the best result was considered valid, each hand was evaluated singularly, time required to complete the task was used as score.

1. Krack P, Pollak P, Limousin P, et al. Subthalamic nucleus or internal pallidal stimulation in young onset Parkinson’s disease. Brain J Neurol. 1998;121 (Pt 3):451–457.

2. Bohnen NI, Kuwabara H, Constantine GM, Mathis CA, Moore RY. Grooved pegboard test as a biomarker of nigrostriatal denervation in Parkinson’s disease. Neurosci Lett. 2007;424:185–189.

Supplementary Figures and Tables

Table S1. UPDRS III values.

MDS - UPDRS part III	T0	T2	ΔT0T2	ΔT0T2%
Real	40.7±10.6	30±10.6	10.7±6.3	27.1±16
M1-PFC	42.4±11.2	30.5±10.3	11.9±6.6	28.6±15.1
M1	39.1±10	29.5±11.2	9.5±6	25.8±17
Sham	43.3±9	36.8±9.6	6.5±7.4	14.6±17.1
Sig. (p-value) Real vs. Sham			*0.027*	*0.007*

Table S1. UPDRS III raw values at baseline and at the end of the active phase of the protocol and their absolute (ΔT0T2) and percentage (ΔT0T2%) change.

Data are shown as average ± standard deviation. P-value is calculated analyzing Real group vs. Sham group.

Table S2. UPDRS III Tremor subscores.

UPDRS III tremor subscore	T0	T2	ΔT0T2	ΔT0T2%
Real	8.5±5.1	6.2±3.7	2.3±3.1	22.9±31
M1-PFC	7.3±3.9	5.2±2.4	2.1±3.4	25.9±30
M1	9.7±5.9	7.2±4.4	2.5±2.9	20.2±32
Sham	6.6±3.2	6.4±3.1	0.2±2.1	1.2±32
Sig. (p-value)Real vs. Sham			*0.005*	*0.001*

Table S2. UPDRS III tremor score at baseline and at the end of the active phase of the protocol and their absolute (ΔT0T2) and percentage (ΔT0T2%) change.

Data are shown as average ± standard deviation. P-value is calculated analyzing Real group vs. Sham group.

Table S3. Timed Tests

HT_WS	T0	T2	ΔT0T2	ΔT0T2%	HT_BS	T0	T2	ΔT0T2	ΔT0T2%
Real	26.3±7.3	30.9±7.6	4.6±5.6	20±23	Real	28.6±7.7	32.3±8.7	3.7±4.4	14±18
M1-PFC	25.8±6.9	30.4±8	4.6±5.6	20±25	M1-PFC	28.8±8.1	32.2±8.6	3.3±3.6	13±16
M1	26.8±7.8	31.4±7.4	4.6±5.8	20±23	M1	28.4±7.5	32.5±9.0	4.1±5.1	15±20
Sham	28.1±7.1	30.4±7.3	2.8±3.2	11±16	Sham	29.1±7.4	31.9±8.6	3.0±5.2	12±21
Sig. (p-value) Real vs. Sham			*ns*	*0.041*	*Sig. (p-value) Real vs. Sham*			*ns*	*ns*
**FT_WS**					**FT_BS**				
Real	27.5±6.4	35.7±8.2	8.2±6.2	32±26	Real	29.3±7.1	36.5±9.3	7.2±5.7	26±21
M1-PFC	26.4±6.6	34.9±10	8.4±7.6	34±30	M1-PFC	28.6±7.9	36.4±11.7	7.8±6.3	27±22
M1	28.4±6.1	36.4±6.5	8.0±4.8	31±21	M1	30.0±6.3	36.7±6.8	6.7±5.2	24±20
Sham	27.4±5.2	31.9±7.8	4.5±5.5	16±20	Sham	28.3±6.6	33.0±7.6	4.7±4.5	18±17
Sig. (p-value) Real vs. Sham			*0.027*	*0.012*	*Sig. (p-value) Real vs. Sham)*			*ns*	*n.s.*
**NHPT_WS**					**NHPT_BS**				
Real	35.7±10.8	31.7±11.8	4.0±5.4	12±14	Real	30.8±10.8	29±9.5	1.7±4.0	5±12
M1-PFC	37.4±11.8	33.8±14.2	3.6±5.4	11±13	M1-PFC	30.4±9.5	30.1±9.5	0.4±3.2	0.5±11
M1	34.2±9.8	29.8±9.1	4.4±5.5	12±14	M1	31.1±12.1	28.2±9.7	2.9±4.5	8±11
Sham	33.5±12.9	33.0±7.8	0.5±6.9	3±18	Sham	30.6±9.7	28.0±5.7	2.5±7.0	5±16
Sig. (p-value) Real vs. Sham			*0.018*	*0.003*	*Sig. (p-value) Real vs. Sham*			*ns*	*n.s.*
**TW**									
Real	34.3±7.3	33.1±7.7	1.2±5.6	2±15					
M1-PFC	33.3±7.4	33.0±7.9	0.33±5.8	0.3±15					
M1	35.2±7.4	33.1±7.7	2.0±5.4	4±15					
Sham	34.3±5.7	33.4±5.8	0.5±4.7	0.2±20					
Sig. (p.value) Real vs. Sham			*ns*	*ns*					

Table S3. Timed tests (Hand Tapping – HT; Foot Tapping – FT; Nine hole peg test - NHPT) scores for the worse and better sides (WS and BS respectively) at baseline and at the end of the active phase of the protocol and their absolute (ΔT0T2) and percentage (ΔT0T2%) change.

Data are shown as average ± standard. P-value is calculated analyzing Real group vs. Sham group.

MMSE	T0	T2	ΔT0T2	ΔT0T2%
Real	27.9±2.4	28.1±2.3	0.1±2.1	1±8
M1-PFC	27.5±2.5	28.2±2.3	0.6±2.2	2±8
M1	28.4±2.2	28.2±2.2	0.3±1.7	1±6
Sham	28.3±1.9	28.7±1.9	0.3±2.1	1±8
Sig. (p-value) Real vs. Sham			*ns*	*ns*
Forward digit span				
Real	5.7±1.8	6.3±1.5	0.5±1.4	14±40
M1-PFC	5.9±1.6	6.5±1.5	0.6±1.3	18±48
M1	5.7±1.8	5.6±1.9	0.2±1.5	5±28
Sham	6.5±1.7	6.7±1.8	0.1±2.2	5±34
Sig. (p-value) Real vs. Sham			*ns*	*ns*
Phonemic fluencies				
Real	31.0±11.6	32.8±11.8	2.9±4.4	16±38
M1-PFC	30.4±12.4	32.9±12.6	3.1±3.7	22±50
M1	31.3±12.0	32.7±10.3	1.9±7.8	10±25
Sham	32.5±9.5	34.6±10.5	1.2±7.1	4±25
Sig. (p-value) Real vs. Sham			*ns*	*ns*
BDI - II				
Real	10.2±7.9	9.0±7.1	1,4±6.2	8±57
M1-PFC	11.7±8.3	10.5±8.2	1.3±7.3	4±70
M1	8.0±7.2	6.7±4.7	1.7±4.9	20±36
Sham	10.7±8.6	9.2±8.9	0.4±4.2	20±150
Sig. (p.value) Real vs. Sham			*ns*	*ns*

FAB	T0	T2	ΔT0T2	ΔT0T2%
Real	13.8±3.7	15.1±2.5	1.2±3.5	22±72
M1-PFC	13.4±3.5	14.7±2.7	1.2±3.4	26±60
M1	14.4±3.4	15.7±2.0	1.2±3.5	16±38
Sham	13.5±2.4	14.6±2.4	0.8±2.6	9±25
Sig. (p-value) Real vs. Sham			*ns*	*ns*
Backward digit span				
Real	4.3±1.5	4.5±1.4	0.1±1.2	8±35
M1-PFC	4.5±1.4	4.3±1.5	0.2±1.1	2±24
M1	4.2±1.6	4.5±1.5	0.1±1.5	15±45
Sham	5.2±1.28	4.8±1.7	0.4±2.2	3±43
Sig. (p-value) Real vs. Sham)			*ns*	*ns*
Semantic fluencies				
Real	40.1±8.6	40.2±11.0	1.1±4.7	3±12
M1-PFC	40.4±8.4	38.5±8.8	-1.5±4.4	1±12
M1	40.8±9.9	41.5±9.9	2.6±4.6	7±11
Sham	40.1±10.5	42.2±10.6	-1.2±5.9	4±18
Sig. (p-value) Real vs. Sham			*ns*	*ns*

Table S4. Neuropsychological tests (MMSE - Mini Mental State Examination; FAB – Frontal Assessment Battery; BDI – Beck Depression Inventory) scores at baseline and at the end of the active phase of the protocol and their absolute (ΔT0T2) and percentage (ΔT0T2%) change. All the data are shown as uncorrected raw values.
Data are shown as average ± standard. P-value is calculated analyzing Real group vs. Sham group.

Figure S1. UPDRS part III worse side subscore improvement at the end of the protocol.

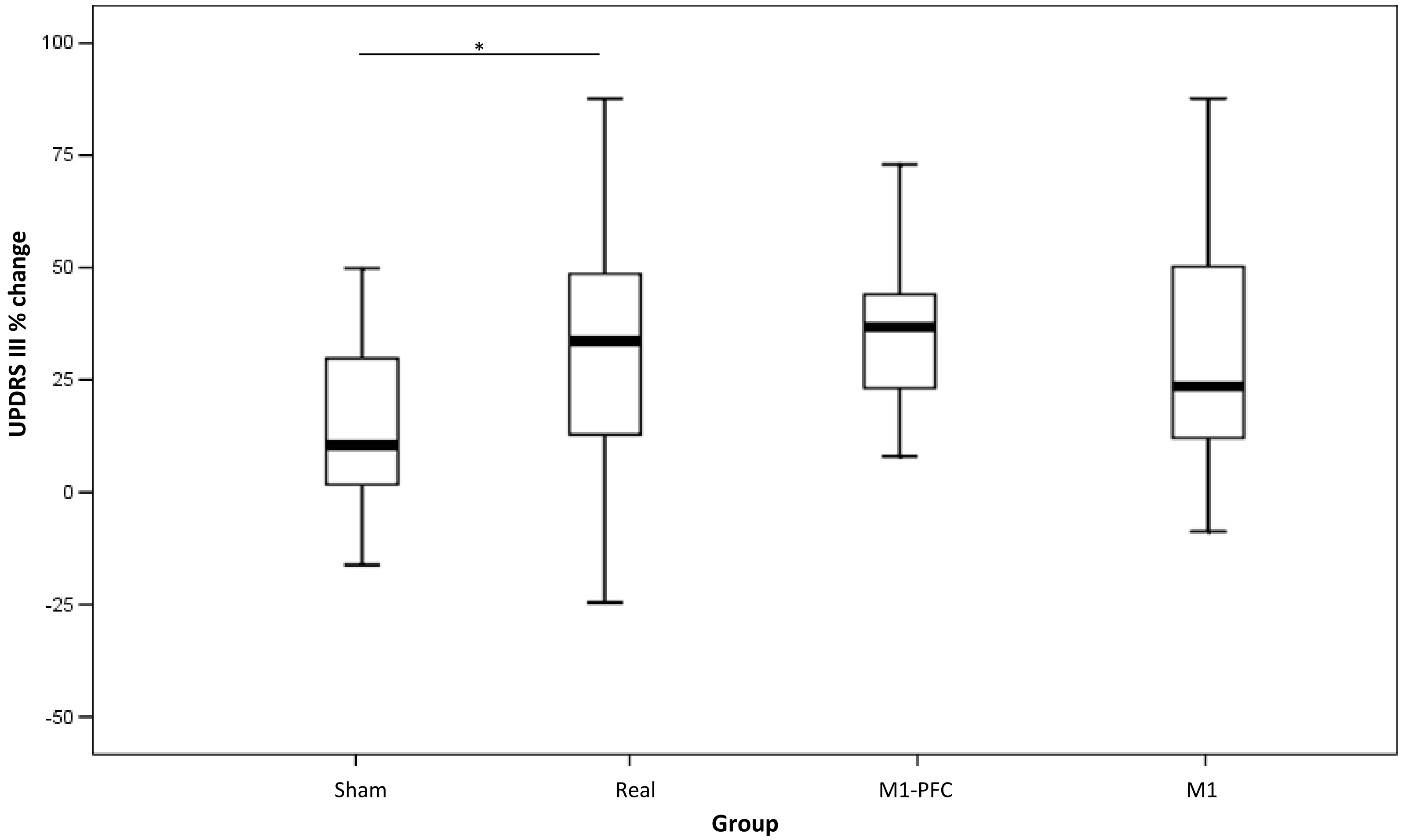

Figure S1. Percent variation of Unified Parkinson's Disease rating scale (UPDRS) part III for the worse side at T2 compared to basal. Real group is obtained by merging M1-PFC and M1 together. T-test, * p<0.05 (Real vs. Sham).

Figure S2. UPDRS part III better side subscore improvement at the end of the protocol.

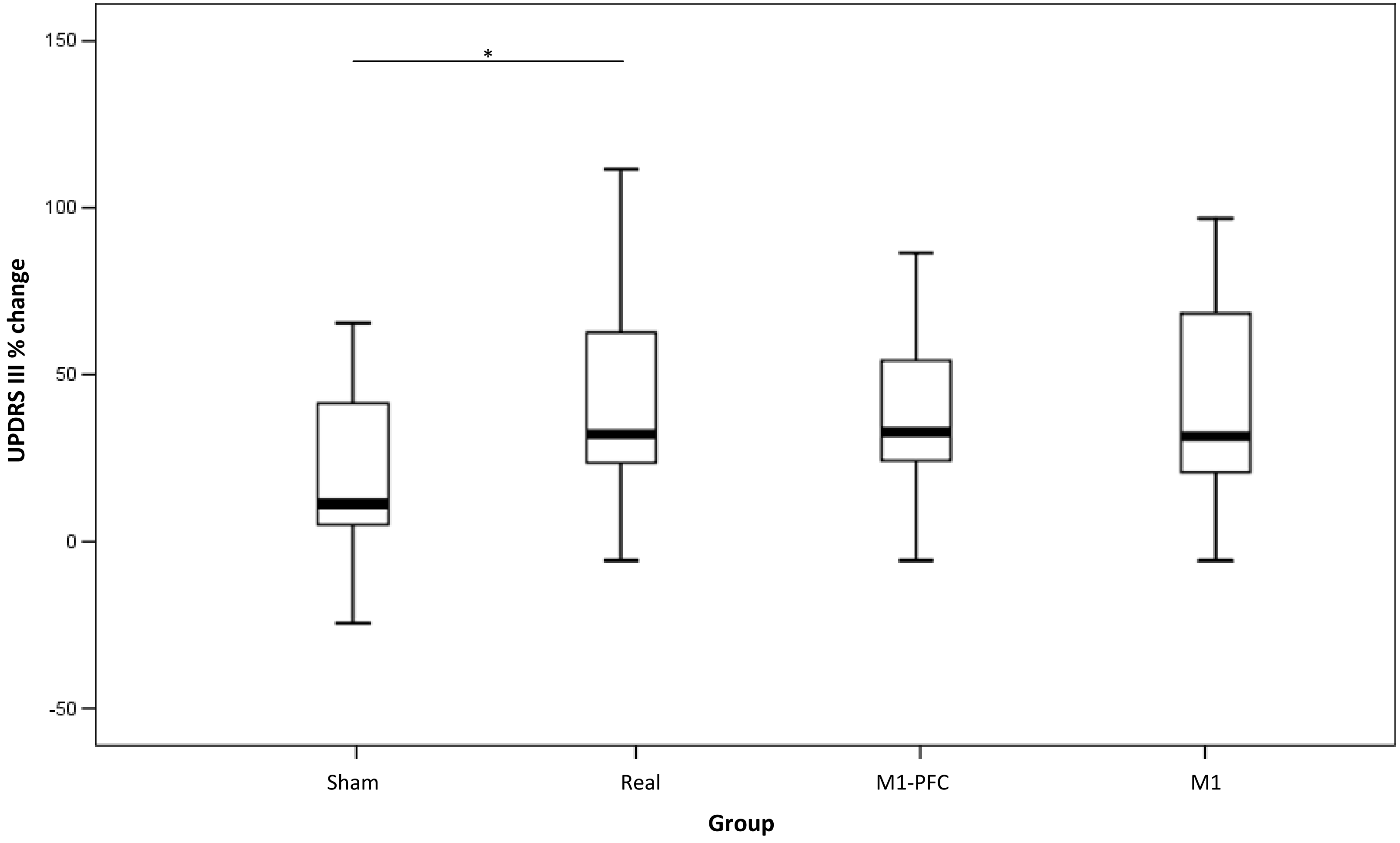

Figure S2 Percent variation of Unified Parkinson's Disease rating scale (UPDRS) part III for the better side at T2 compared to basal. Real group is obtained by merging M1-PFC and M1 together. T-test, * $p<0.05$ (Real vs. Sham).

Figure S3. Hand tapping improvement at the end of the protocol.

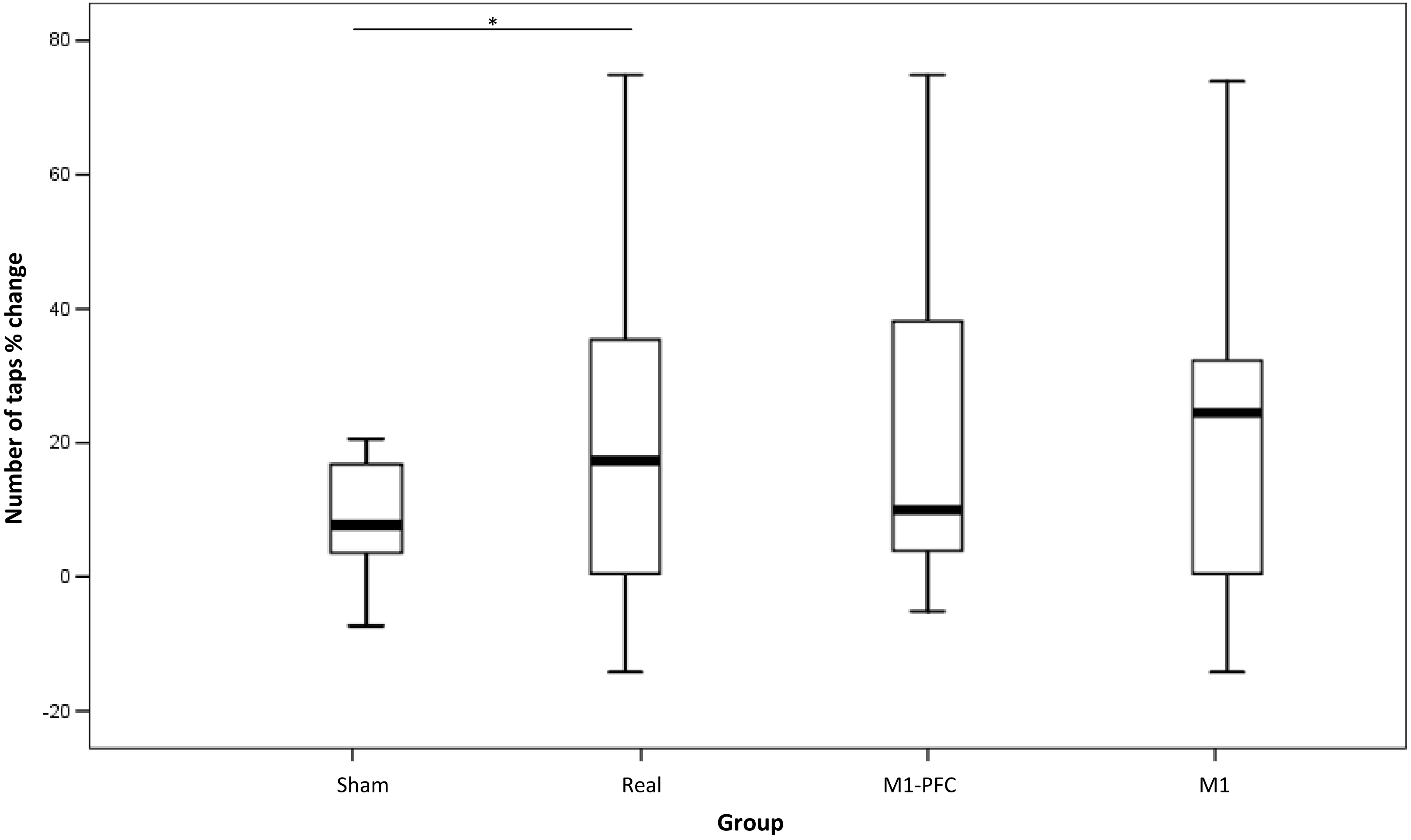

Figure S3. Percent variation for the hand tapping (HT) for the worse side at T2 compared to basal. Real group is obtained by merging M1-PFC and M1 together. T-test, * $p<0.05$ (Real vs. Sham).

Figure S4. Foot tapping improvement at the end of the protocol.

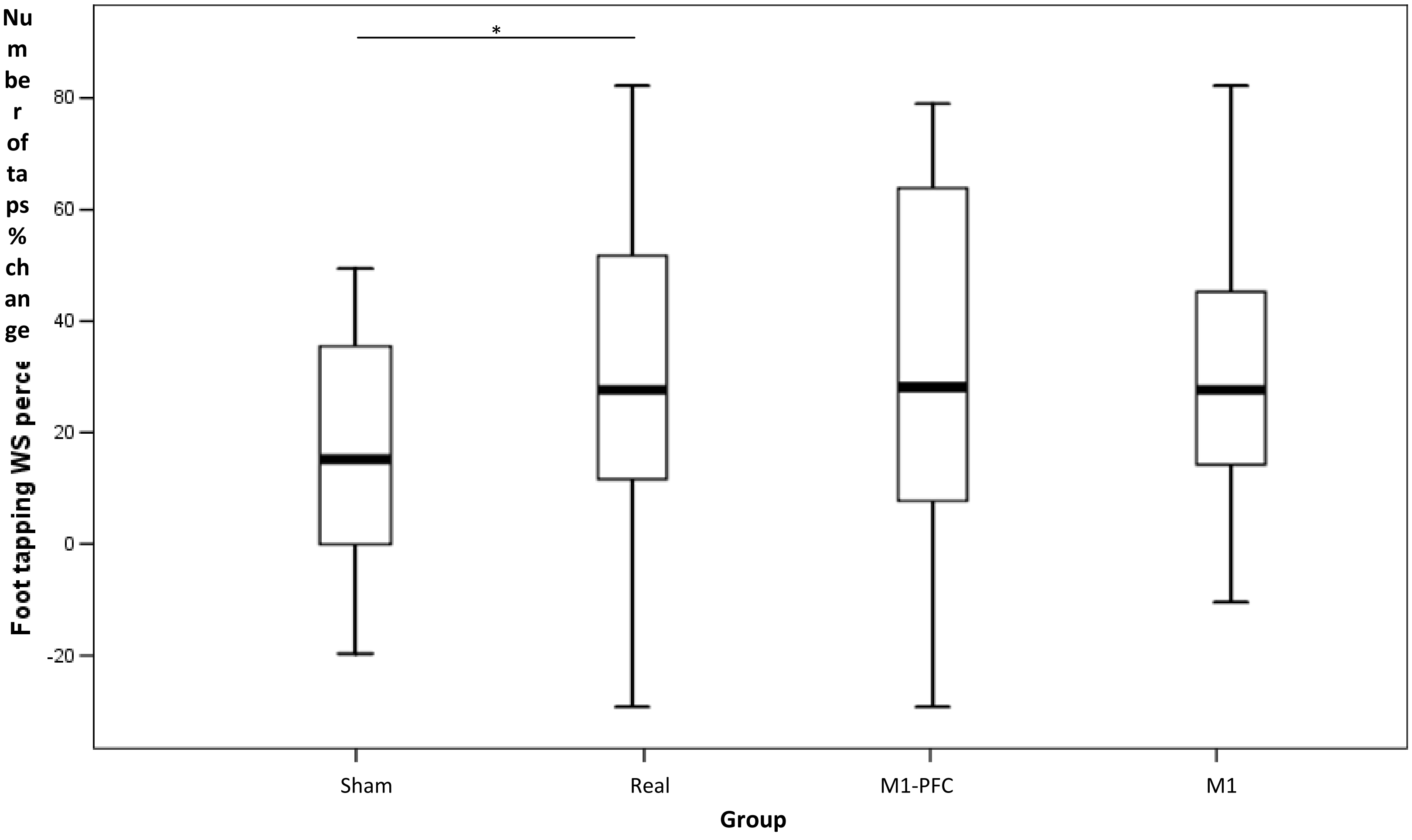

Figure S4. Percent variation for the foot tapping (FT) for the worse side at T2 compared to basal. Real group is obtained by merging M1-PFC and M1 together. T-test, * $p<0.05$ (Real vs. Sham).

Figure S5. Nine Hole Peg Test improvement at the end of the protocol.

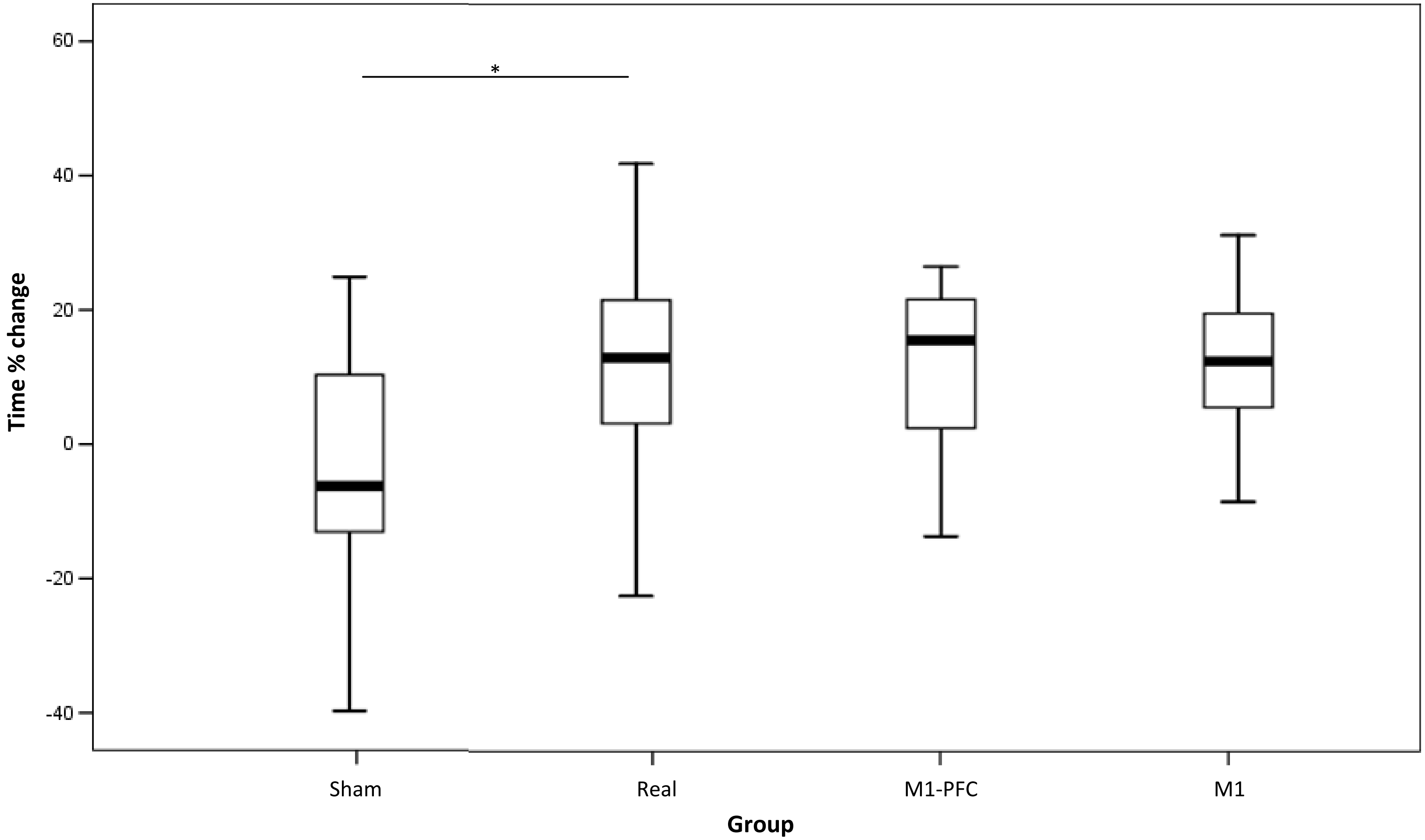

Figure S5. Percent variation for the nine hole peg test (NHPT) for the worse side at T2 compared to basal. Real group is obtained by merging M1-PFC and M1 together. T-test, * $p<0.05$ (Real vs. Sham).
